# Ocular injuries in survivors of improvised explosive devices (IED) in commuter trains

**DOI:** 10.1186/1471-227X-7-16

**Published:** 2007-09-27

**Authors:** Salil Mehta, Vinay Agarwal, Prakash Jiandani

**Affiliations:** 1 Dept of Ophthalmology, Lilavati Hospital and Research Centre, Mumbai, India; 2 Dept of Critical Care Medicine, Lilavati Hospital and Research Centre, Mumbai, India

## Abstract

**Background:**

Ocular injuries are common in survivors of terror incidents that involve the use of explosive materials. These explosives are commonly of a High Explosive type (HE) and may be fashioned into improvised explosive devices (IED) that incorporate additional materials to maximise trauma and injuries. Serial IED explosions have occurred in commuter trains in several cities including London and Madrid but data on ocular injuries is limited. We report the ocular injuries of the survivors of a series of IED explosions in crowded commuter trains.

**Methods:**

28 patients (56 eyes, 28 male, ages ranging from 22 to 52 years (mean 35.27 years) were screened in the triage area or the Intensive Care Unit (ICU). Testing included bedside visual acuity testing, torchlight examination of the anterior segment and dilated (or if necessary, undilated) fundus examination. Selected patients underwent B-scan examination, magnetic resonance imaging of the brain, orbits and the optic nerves or visual evoked potential assessment. The injuries, investigations and procedures were entered into the patient's case sheet as well as into a standardised format suggested by the Indian eye injury registry (IER).

**Results:**

16 of 28 patients (57.1%) had ocular injuries whereas 12 (42.8%) were found to be normal. Injuries were seen unilaterally in 10 patients and bilaterally in six yielding a total of 22 injured eyes. The common injuries were periorbital haemorrhages (09 eyes, 40%); first or second degree burns to the upper or lower lids (seen in 07 eyes, 31.8 %) and corneal injuries (seen in 08 eyes, 36.3%). Open globe injuries were seen in two eyes of two patients (09%). One patient (4.5%) had a traumatic optic neuropathy.

**Conclusion:**

Ophthalmologists and traumatologists should be aware of these patterns of ocular injuries. Protocols need to include the screening of large numbers of patients in a short time, diagnostic tests (B scan, visual evoked potential (VEP) etc) and early surgery preferably at the initial triage itself as most of the serious injuries in our studies had been missed or not treated at an initial assessment.

## Background

Ocular injuries are increasingly being recognized in association with terrorist acts and may be associated with anatomical and functional morbidity. Commonly used explosives are categorized as high-order explosives (HE) or low-order explosives (LE). HEs produce a characteristic over-pressurization shock wave and include TNT, C-4, Semtex and dynamite. LEs create a subsonic explosion that differs significantly from HE in its impact. HE may be used either as either regular munitions or fashioned into improvised explosive devices (IED) that consist of a core of HE with associated materials including oil and shrapnel that increase casualties [1]. Most recent incidents worldwide have been carried out with the use of IEDs including those in Kashmir, Iraq and Israel.

Despite the fact that the eye is relatively small, being responsible for only 0.1% of the frontal surface area, ocular injuries are common and may be seen in 3–10% of survivors of terrorist blasts [2].

Regardless of the type, all explosions cause injuries by numerous means. The initial event is a positive phase that produces a massive increase in atmospheric pressure (blast wave) followed by a negative phase in which the drop of atmospheric pressure pulls debris into the blast area and a blast wind. The initial blast wave produces the injuries to the lungs, gastrointestinal system, central nervous system and the eardrums (primary injuries). Secondary injuries include those caused by glass and masonry that is ejected by the explosion. Tertiary injuries include those caused by the blast wind. Thermal injuries are also common and are caused by an initial brief high temperature. The pattern and prevalence of ocular injuries depends on several factors including IED composition, the setting of the blast, the location of the victim relative to the blast and the availability of medical aid.

We report the ocular injuries of 28 patients who were survivors of serial IED explosions in commuter trains.

## Methods

This is an Institutional review board approved retrospective non-comparative study. A series of IED explosions in five crowded local commuter trains occurred on a single day leaving at least 190 dead and at least 700 injured. The injured were immediately shifted to various surrounding hospitals and following stabilisation, 41 patients were transferred to our hospital. A detailed evaluation was done including clinical examination, blood work-up and radiographic studies of the chest, abdomen and head.

All patients with suspect ocular injuries (injuries in the head or face areas) were screened in the triage area or the intensive care unit. All underwent bedside visual acuity testing (finger counting, if possible), torchlight examination of the anterior segment and dilated fundus examination with the indirect ophthalmoscope. Dilatation of the pupils was not carried out if preservation of the pupillary reflex was required for neurological monitoring or they had open globe injuries and this test was completed in these patients prior to discharge. Patients with globe injuries or external foreign bodies underwent B-scan to rule out retained intraocular foreign bodies. Patients with complaints of visual loss without obvious globe injuries or with abnormal pupillary reactions, specifically relative afferent pupillary defects (RAPD) underwent magnetic resonance imaging of the brain, orbits and the optic nerves and visual evoked potential assessment (once stable) to rule out traumatic optic neuropathy. All patients who underwent treatment (conservative or operative) were monitored daily till discharge. At discharge, patients were re-evaluated including slit lamp examination, vision testing and dilated fundus examination if not done earlier. The injuries, investigations and treatment offered were entered into the patient's case sheet as well as into a standardised format suggested by the Indian eye injury registry (IER) [3].

## Results

28 patients ((56 eyes, 28 male, ages ranging from 22 to 52 years (mean 35.27 years)), with suspect ocular injuries (injuries in the head or face areas) were screened in the triage area or the intensive care unit. All underwent the examination described earlier. Four patients (14.2%) underwent only undilated fundus examination, as the preservation of the pupillary reflex was required for neurological monitoring (n = 2, 7.1%) or they had open globe injuries (n = 2, 7.1%). Three patients (10.7%), those with globe injuries or external foreign bodies underwent B-scan to rule out retained intraocular foreign bodies. Two patients (7.1%) with complaints of visual loss without obvious globe injuries or with abnormal pupillary reactions, specifically relative afferent pupillary defects (RAPD) underwent magnetic resonance imaging of the brain, orbits and the optic nerves and visual evoked potential assessment (once stable).

On initial examination, 16 of 28 patients (16 male, 57.1%) had ocular injuries whereas 12 (42.8%) were found to be normal. Injuries were seen unilaterally in 10 patients and bilaterally in six yielding a total of 22 injured eyes. The various ocular injuries noted are detailed in table 1. The common injuries were periorbital haemorrhages (09 eyes, 40%); first or second degree burns to the upper or lower lids (seen in 07 eyes, 31.8 %) and corneal injuries (seen in 08 eyes, 36.3%). Open globe injuries were seen in two eyes of two patients (09%, ages 33 and 36 years). One patient had external impacted foreign body with no intraocular injury. One patient (4.5%) had traumatic optic neuropathy that presented as visual loss with a relative afferent pupillary defect (RAPD) and was later confirmed on visual evoked potential testing. Overall, the commonest structure involved were the eyelids with 11 eyes (50%) showing only lid burns or haemorrhages and another four eyes (18.1%) showing lid and corneal injuries

**Table 1:** Showing the various ocular injuries in survivors

**Findings**	**Number of eyes (N = 22)**	**Percentage of total**
Lid Burns	07	31.8
Corneal Injuries	08	36.3
Corneoscleral Tears	02	09.0
Periocular haemorrhages	09	40.0
Traumatic optic neuropathy	01	04.5

Total	27 *	121.6 *

The total exceeds 22 (100%) as some eyes had more than one finding

Open globe injuries were seen in two eyes of two patients (09%). Of these, one patient (33, male) had an open globe injury of the right eye with a corneoscleral tear (7 mm) with iris prolapse. There was no evidence of retained intraocular foreign body. The other patient (36, male) had a severely injured left eye with an extensive 14 mm corneoscleral tear with iris prolapse, lens extrusion, and vitreous loss. One patient (4.5%) had an external impacted foreign body with no apparent intraocular injury.

These three patients required operative intervention. Preoperative visual acuity testing was not possible in these patients due to poor overall status and high injury severity scores and they underwent ocular surgery in conjunction with orthopaedic or surgical procedures. The patient with right eye injury underwent primary repair with iris repositioning. There were no postoperative complications and visual acuity was 6/9 on day 7 and a B scan revealed a normal posterior segment. The patient with the left eye injury underwent primary corneal repair, iris abscission and an open sky vitrectomy. A metallic foreign body of approximately 1–2 mm was removed from the anterior chamber angle. On day 7, his visual acuity was no perception of light in the affected eye and a B scan revealed total retinal detachment with closed funnel configuration. The patient with the external foreign body underwent removal with forceps with no postoperative complications (post operative vision was 6/6 in the affected eye).

The patient with the traumatic optic neuropathy underwent a regimen of intravenous methylprednisolone (1 gm/day) for seven days. A MRI scan revealed normal globes and orbits and diffuse axonal injury of the brain with a VEP suggestive of left sided optic nerve injury. He had a good visual outcome (6/6 bilaterally).

The remaining patients were conservatively managed with topical lubricants, antibiotics and corticosteroid drops. There was no ocular morbidity in any of these patients at discharge.

Severe systemic injuries requiring extensive surgery were seen in the two patients with open globe injuries. These included amputation of a severely traumatised arm, exploratory abdominal surgery and severe body burns. Other patients with less severe ocular injuries had extensive burns, lung contusions and blunt abdominal injuries.

## Discussion

In 2005–2006, worldwide terror incidents were estimated by different sources to range from 5023 to 11,500 with an estimated 8364 to 14,500 fatalities [4].

Differing patterns of ocular injuries have been reported in various terror incidents and depend on the construction of the IED, the location of the victim relative to the blast and the setting of the blast. The crowded commuter train presents a unique milieu both in terms of crowd density as well as being a closed environment. According to media reports, the explosive devices used were later to found to consist of a core of RDX (Research Development Explosive) with a surrounding layer of a mixture of ammonium nitrate and fuel oil (probably diesel oil) and packed within a pressure cooker [5,6]. Ammonium nitrate is a strong oxidising agent and forms an explosive mixture when used in a mixture with fuel oil (AN-FO).

In our series, the predominant injuries were minor and non-vision threatening injuries of the anterior segment, with as many as 19 of the 56 eyes (33.9 %) we examined showing such lid, conjunctival, corneal injuries or periocular hemorrhages. This finding may be due to the construction of the IEDs used in this incident, specifically the RDX/AN-FO mixture that caused primarily overpressure and burn injuries rather than shrapnel-induced penetrating injuries. Three eyes had more severe injuries with two patients having penetrating injuries and one with traumatic optic neuropathy. The open globe injuries may be due to either a direct blast effect that produces tissue damage across an air-fluid interface or were due to projectile injury. A projectile injury was likely in the patient with the retained intraocular foreign body. The traumatic optic neuropathy is likely to be due to non-blast causes and may include injuries caused by falls or collapsing walls and roofs. We placed a greater reliance on clinical examination and had to defer definitive investigations in our patients as they were severely injured and first priority was accorded to life saving systemic surgery.

The patients we studied were transferred to our institute by their next of kin or relatives to seek better treatment, possibly creating a bias towards less severe systemic injuries and our results may not represent the entire spectrum of ocular injuries in these patients. Collection of more data, especially from primary treatment centers, may provide a more accurate picture of the ophthalmologic impact of this incident. All the injuries that needed specialized ocular treatment (whether surgical or medical) had been missed or not treated at the initial triage. This may potentially impact the future visual outcome in these types of patients.

Earlier multiple coordinated IED attacks on commuter trains were seen in Madrid (2004) and in London (2005). In the Madrid incidents, four of 91 hospitalized patients (%), had severe ocular injuries. These included one traumatic unilateral enucleation, two eyes with intraocular hemorrhage, and two globe perforations with an intraocular foreign body. All these cases resulted in partial or total visual loss [7]. It is not known how many had eyelid or other anterior segment injuries.

Similar ocular injuries are commonly encountered in military or counter-insurgency operations where the use of IEDs is common. IEDs caused 51% of severe ocular and adnexal injuries in a series of 207 patients treated at a U.S. military hospital in Iraq. Common injuries included open globe injuries (132 eyes), lid/brow lacerations (60 eyes) and others with hyphema, vitreous haemorrhage, and intraocular foreign bodies [8].

Ocular and facial injuries in survivors of truck and car bombings may differ due to larger explosive loads and larger distances from the primary blast. 55 of 684 (8%) survivors of the Alfred P. Murrah building (Oklahoma City, Oklahoma, 1995) that was demolished by a parked truck with 4000–5000 pounds of ammonium nitrate had ocular injuries. These included lid/brow lacerations (23 eyes of 20 patients), open globe injuries (12 eyes), orbital fractures (6 eyes) and retinal detachment (5 eyes). These injuries were largely due to the blast wave itself or due to flying glass and debris [9]. Falling masonry is also a risk factor in injury causation in these patients. Similarly, 27.6% of truck bomb survivors of the U.S. Embassy in Nairobi, Kenya had severe eye injuries [10].

Blast ocular injuries are common in firework accidents and may affect any ocular tissue. Sparklers, cones and other light emitting fireworks may cause anterior segment injuries and noise producing devices (bombs etc) potentially may cause damage to both anterior and posterior segments. In one series of eight seriously injured patients, six (75%) showed anterior segment (lid/conjunctival/corneal) burns and another five (62.5 %) had evidence of hyphema amongst other injuries [11]. Four (50%) of these patients had moderate to severe visual loss at follow-up. In another series of 42 patients from India, corneal injuries were seen in 16 (38.5%) patients, corneal foreign bodies in 15 (35.7%) patients), hyphema in 14 (33.3%) patients and open globe injuries in 3 (7.1%) patients [12]. As many as 13 of these 42 patients (30.9%) had moderate to severe visual loss at 3 month follow-up with corneal scarring, vitreous haemorrhage, and macular lesions being responsible for the majority of these. Sacu et al have reported that skin and corneal injuries were the most common in their series of 116 eyes and were seen in 32 (28%) of them with as many as 11 of the 116 eyes (10%) having severe injuries. 32 of these 116 patients (28%) had permanent visual loss either due to corneal or retinal lesions. In our series, anterior segment burns and injuries are the most common injuries similar to those seen in firework injuries. However several contrasts with firework and other blast injuries remain. The demographics of our patients were different (all adult males) as a consequence of the site and timing of the blasts (commuter trains at evening rush hour). We did not record hyphema in any of our patients. Moderate to severe visual loss was noted in only one patient. This may be a result of the lack of proximity to the blast that also allowed survival of these patients.

The phenomenon of finding mostly non-life threatening conditions in terror attack survivors is well known for systemic injuries but not enough data exists to permit generalization of ocular injuries in similar incidents.

Emergency management planners and emergency physicians should be aware of these patterns of ocular injuries. An ideal in-place protocol would:

1. Activate on-call physicians, primarily trauma surgeons that would conduct primary triage on the pool of injured patients. Ideally all or at least those patients with head/face injuries would undergo further evaluation by further team of eye, ear, nose and throat (ENT), maxillofacial surgeons and neurosurgeons.

2. As a part of this team ophthalmologists should screen all these patients. This should be prior to any surgical intervention except immediate life saving measures like tracheostomies or intercostal drains. A primary division into medical and surgical cases would be an initial step. This would allow ophthalmologists to co-ordinate surgery with other specialities to obviate the need for staggered surgeries and repeated administration of anaesthetic agents. Apart for a detailed clinical examination to the extent possible, a proportion of patients will need diagnostic tests of which computed tomography (to identify orbital fractures and potential optic nerve injuries) and B-scan (to identify intraocular injuries). The ocular surgeries likely to be performed are open globe repairs with or without intraocular foreign body removal. A proportion is likely to require exploratory surgery to assess the exact nature and nature of injuries.

## Conclusion

Ophthalmologists and traumatologists should be aware of the potential for ocular morbidity in survivors of IED attacks. These are likely to be thermal injuries usually affecting the anterior segment though a proportion may have serious vision threatening injuries that may need operative intervention. Early evaluation is needed and protocols need to be developed to allow for the screening of large numbers of patients in a short time, performing the necessary diagnostic tests (B scan, visual evoked potential (VEP) etc) and early surgery. Adoption of such a protocol would allow early specific treatment and minimise the risk of missed ocular injuries.

## Competing interests

The author(s) declare that they have no competing interests.

## Authors' contributions

Dr SM conceived of, collected the data, analyzed and wrote the paper.

Dr VA helped in the data collection, analysis and writing of the paper

Dr PJ helped in the writing and critical review.

All the authors have read and approved the final version of this manuscript

## Pre-publication history

The pre-publication history for this paper can be accessed here:


